# A BMI-based occupational therapy assist suit: asynchronous control by SSVEP

**DOI:** 10.3389/fnins.2013.00172

**Published:** 2013-09-23

**Authors:** Takeshi Sakurada, Toshihiro Kawase, Kouji Takano, Tomoaki Komatsu, Kenji Kansaku

**Affiliations:** Systems Neuroscience Section, Department of Rehabilitation for Brain Functions, Research Institute of National Rehabilitation Center for Persons with DisabilitiesTokorozawa, Japan

**Keywords:** BMI, BCI, SSVEP, exoskeleton, asynchronous control

## Abstract

A brain-machine interface (BMI) is an interface technology that uses neurophysiological signals from the brain to control external machines. Recent invasive BMI technologies have succeeded in the asynchronous control of robot arms for a useful series of actions, such as reaching and grasping. In this study, we developed non-invasive BMI technologies aiming to make such useful movements using the subject's own hands by preparing a BMI-based occupational therapy assist suit (BOTAS). We prepared a pre-recorded series of useful actions—a grasping-a-ball movement and a carrying-the-ball movement—and added asynchronous control using steady-state visual evoked potential (SSVEP) signals. A SSVEP signal was used to trigger the grasping-a-ball movement and another SSVEP signal was used to trigger the carrying-the-ball movement. A support vector machine was used to classify EEG signals recorded from the visual cortex (Oz) in real time. Untrained, able-bodied participants (*n* = 12) operated the system successfully. Classification accuracy and time required for SSVEP detection were ~88% and 3 s, respectively. We further recruited three patients with upper cervical spinal cord injuries (SCIs); they also succeeded in operating the system without training. These data suggest that our BOTAS system is potentially useful in terms of rehabilitation of patients with upper limb disabilities.

## Introduction

Recent advances in robot technologies have facilitated development of new devices to assist the movements involved in rehabilitation training for people with motor dysfunction. The proposed devices use various methods to assist upper limb movements: HEXORR was designed to assist all digits of the hand (Schabowsky et al., 2010), MIT-MANUS can support movements of the elbow and shoulder (Finley et al., 2005) or wrist (Krebs et al., 2007) during planar reaching tasks. Pneu-WREX can supply active forces generated by a pneumatic actuator to support movements of the arm in three-dimensional space (Wolbrecht et al., 2008), and ARMin II has high degrees of freedom (DOFs) for the shoulder, elbow, and wrist to perform coordinated movements associated with activities of daily living (Staubli et al., 2009). Some studies have used robot-assisted rehabilitation in stroke patients (Hesse et al., 2008; Marchal-Crespo and Reinkensmeyer, 2009; Masiero et al., 2011). Goal-directed movement has been suggested to be of value in rehabilitation training (Ma and Trombly, 2002; Pillastrini et al., 2008). The use of robot-assisted rehabilitation in persons with physical disabilities would be enhanced if the system supported goal-directed actions involving multiple body parts; for example, the whole arm, including the fingers. Devices with such movements would be useful in occupational therapy (OT) training. In general, rehabilitation training often requires that the assistive robots exhibit high DOFs to support various goal-directed movements of the upper limbs. However, robot systems developed to date do not sufficiently support the delicate movements of the whole arm, especially the fingers.

These devices can be controlled by physiological signals. For example, electromyography has been used to assist in reaching movements with MIT-MANUS (Dipietro et al., 2005) and elbow movements (Song et al., 2008). Furthermore, these devices can be combined with a brain-machine interface (BMI)/brain-computer interface (BCI), an interface technology that uses neurophysiological signals from the brain to control external machines or computers (Wolpaw et al., 2002; Birbaumer and Cohen, 2007; Kansaku, 2011). Recent invasive BMI technologies have succeeded in the asynchronous control of robot arms for useful series of actions, such as reaching and grasping (Hochberg et al., 2012). Several studies have applied non-invasive BMI technologies to control assistive robots according to user intention (Muller-Putz and Pfurtscheller, 2008; Horki et al., 2010, 2011; Pfurtscheller et al., 2010b; Ortner et al., 2011). These robot-assisted therapies, which use either invasive or non-invasive BMI systems, are an attractive approach to recovery of motor dysfunction in neurorehabilitation (Marchal-Crespo and Reinkensmeyer, 2009; Pignolo, 2009). A BMI-based assistive robot can construct an artificial neurological closed-loop between the brain and end effectors, such as the hands or legs; this closed-loop enhances the plastic changes in the brain during rehabilitation training (Lebedev and Nicolelis, 2006). Recent studies have shown that neural activity may change after invasive (Collinger et al., 2013) or non-invasive BMI (Pichiorri et al., 2011) training. Together, the data suggest that goal-directed actions using BMI technology are potentially of value.

Proposed BMI systems have used electroencephalogram (EEG) signals, elicited by motor imagery, such as event-related de-synchronization (ERD) (Allison et al., 2010; Gomez-Rodriguez et al., 2011; Horki et al., 2011) or by visual stimuli, such as P300 (Farwell and Donchin, 1988; Wolpaw et al., 2002) or steady-state visual evoked potential (SSVEP) (Zhu et al., 2010). Because non-invasive BMI systems do not require surgery to implant electrode(s), as in invasive BMI, these technologies can also be applied to many patients, safely and easily. Furthermore, because control of the external devices of SSVEP- and P300-based BMI systems requires less training than do motor imagery ERD-based systems, such BMIs using visual stimuli are beneficial for people with disabilities in that they can be used immediately. Moreover, SSVEP signals may be detected using a single electrode of a BMI system (Luo and Sullivan, 2010), and SSVEP is thus potentially valuable for use in practical BMIs.

SSVEP can be observed mainly from the visual cortex when a person is focusing visual attention on a flickering stimulus and can be modulated at a frequency higher than 6 Hz (Regan, 1989; Pastor et al., 2003). This is the same fundamental frequency as that of the flickering stimulus, as well as its harmonics. Several studies have applied SSVEP-based BMI to operating tools for living environments (Cheng et al., 2002; Wang et al., 2006), a mouse cursor (Trejo et al., 2006; Diez et al., 2011; Volosyak, 2011; Wilson and Palaniappan, 2011), and a wheelchair (Muller et al., 2010; Bastos et al., 2011). BMI systems using SSVEP signals have the advantage that there is no need to control the timing of stimulus presentation. Thus, BMI users can control the external device asynchronously, depending on their intentions.

In this study, we developed non-invasive BMI technologies to facilitate useful movements through the subject's own hands by preparing a BMI-based occupational therapy assist suit (BOTAS). BOTAS has high DOFs to assist whole upper limb movements, including those of the fingers, and can conduct various types of movement, such as goal-directed movements, during OT. We prepared pre-recorded series of useful actions, a grasping-a-ball movement and a carrying-the-ball movement, and added asynchronous control using SSVEP signals. A SSVEP signal was used to trigger the grasping-a-ball movement and another SSVEP signal was used to trigger the carrying-the-ball movement. Participants were asked to fixate on LED flickers when they sought to start pre-recorded movements. We describe such sequential movements as a “BOTAS-assisted trial” in this study. A support vector machine (SVM) was used to classify the EEG signals recorded from the visual cortex (Oz) in real time. By doing so, we showed that able-bodied participants and patients with upper cervical spinal cord injuries (SCIs), with no previous training, could operate the BOTAS system successfully.

## Materials and methods

### A BOTAS system based on SSVEP

**Figure 1:**
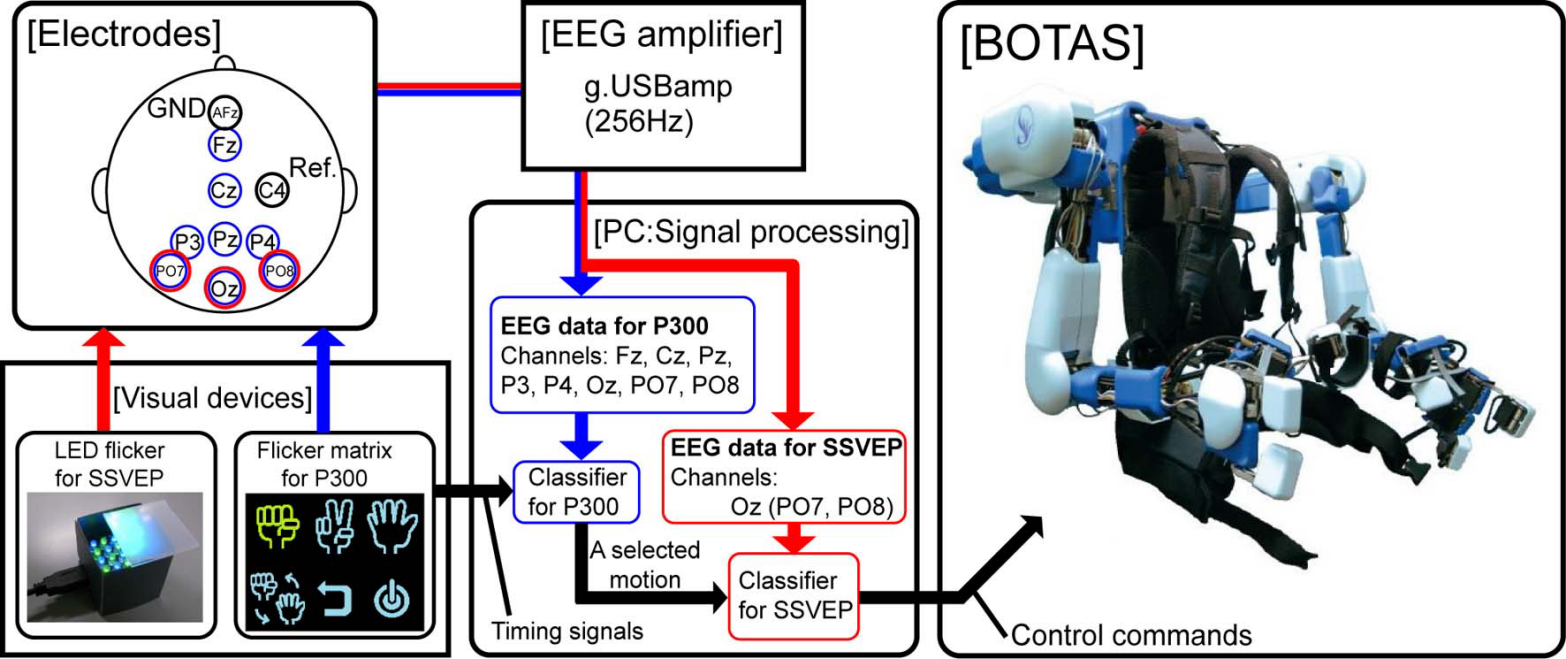
**System overview.** The red arrow indicates the flow of the SSVEP-based BCI system and the blue arrow indicates the flow of a combined BCI system based on both P300 and SSVEP. In this study, we focused on use of the SSVEP-based BCI system to control upper limb movements asynchronously by means of BOTAS.

Figure 1 shows a schematic of our BMI system for BOTAS control. The BOTAS system consisted of a PC, EEG electrodes, EEG cap, amplifier, visual devices, and the assist suit robot. Recently, some studies of hybrid BMIs have appeared (Pfurtscheller et al., 2010a). One work employed a combined ERD-plus-SSVEP system (Allison et al., 2010; Horki et al., 2011) and another a P300-plus-SSVEP system (Panicker et al., 2011). In our system, BOTAS can also be controlled using a hybrid BMI system with SSVEP and P300 (blue arrow procedure in Figure 1) (Sakurada et al., 2011). Our group previously used the so-called P300 speller (Farwell and Donchin, 1988) in a BMI system (Ikegami et al., 2011; Takano et al., 2011); we also included the P300 procedure in the BOTAS system. A monitor displays a flicker matrix and each flickering icon indicates a BOTAS motion that was recorded beforehand. BOTAS users can select a motion using the P300 procedure and can control the initiation of the selected motion using the SSVEP procedure. A hybrid system would be particularly useful when the BOTAS user has to choose a motion from many options (i.e., motions that were recorded beforehand). In this study, to discuss use of asynchronous control systems for a specific task, we focused on the SSVEP-based BMI system.

We prepared visual flicker devices in which green and blue LEDs were arranged in a checkerboard pattern and each color LED flickered alternately at the same frequency. The flickering stimuli afforded by the LEDs induced SSVEP around the visual cortex. Buffered EEG signals (3 s), recorded from PO7, Oz, and PO8 sent classification process information every 0.1 s. We conducted fast Fourier transformation (FFT) and canonical correlation analysis (CCA) of the buffered EEG signals (Bin et al., 2009). Results of the FFT and CCA were used for classification by SVM. Finally, the PC (i.e., the BOTAS controller) sent control commands to BOTAS according to the classification result. Because each LED flicker was assigned to an upper limb motion that was recorded beforehand, BOTAS wearers could select and initiate a motion to assist their own movement at any time. In the next section, we provide further details of each component of the BOTAS system.

#### BOTAS specifications

**Figure 2:**
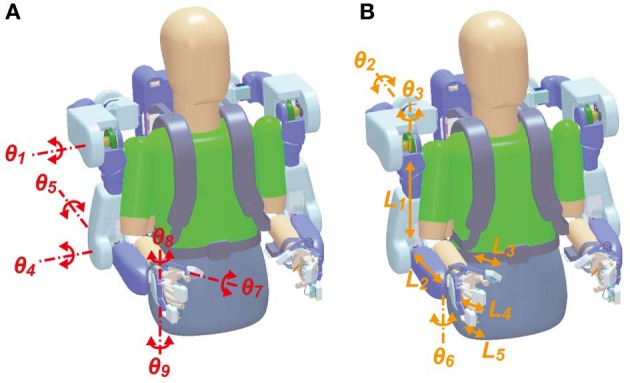
**DOFs in the BOTAS hardware. (A)** Joints of the shoulder, elbow, wrist, and fingers were driven by actuators according to control commands (θ_1_, θ_4_, θ_5_, θ_7_, θ_8_, and θ_9_). **(B)** According to participant physical size or task requirement, the angles in the shoulder joint (θ_2_ and θ_3_) and the wrist joint (θ_6_) and the link length of the upper arm, forearm, and fingers (*L*_1_–*L*_5_) can be adjusted.

BOTAS has six DOFs in each robot-arm to assist with various upper limb movements (Figure 2A): one DOF in each shoulder joint (θ_1_: extension—flexion), one in each elbow joint (θ_4_: extension—flexion), one in each wrist joint (θ_5_: adduction—abduction), and three DOFs in the finger joints of each hand (θ_7_–θ_9_: extension—flexion for thumb, index and middle, and annular and little). DC servomotors were used to drive these joints, except the wrist joint, which was achieved with Bowden cables. The adduction-abduction movements of the wrist joint were driven directly by a DC servomotor. Additionally, the angles of the shoulder's adduction-abduction (θ_2_), internal-external rotation (θ_3_) and the wrist's extension-flexion (θ_6_) were adjusted and fixed according to the posture required in the various tasks (Figure 2B). The system comprises nine DOFs, in total, in each robot arm. Therefore, not only the movements made in the present study (i.e., grasping and reaching movements; see Performance Evaluation of the BOTAS System) but also other movements may be assisted because BOTAS is associated with multiple DOFs, as described above. The link lengths of the BOTAS upper arm, forearm, and fingers are also adjustable (*L*_1_–*L*_5_). When a participant wears the BOTAS, she/he rests her/his elbows on small boards attached to the left and right elbow joints, and her/his forearms, wrists, palms, and fingers are fastened using Velcro fasteners.

To ensure the safety of the wearer, BOTAS can move only within the space defined by the range of motion (ROM), which is measured individually. We checked the ROM of the shoulder, elbow, wrist, and fingers before starting the tasks. Maximum angular velocities were 1.57 rad/s for fingers and 1.05 rad/s for the other joints. When BOTAS assists a grasping or pinching movement, the maximum support power for the finger is 11 N. If a wearer receives overload from BOTAS during movement, the Bowden cables that drive the BOTAS joints cut automatically. Additionally, the BOTAS system can be stopped at any time by pressing an emergency button. Operators (e.g., an experimentalist or a therapist) were asked to press the emergency button when necessary.

BOTAS can be driven and controlled by two methods. In the first, a specified joint is controlled by commands that require it to move in an arbitrary angle. This is effective if the wearer wants to control her/his posture freely, assisted by BOTAS. In the other method, the BOTAS system runs a recorded motion, which is registered in system memory beforehand. The maximum number of recorded motions is eight. This replaying and repeating a recorded motion is useful when a wearer is required to perform a repetitive movement or task, such as in rehabilitation training. In our tasks, we used the latter method to control BOTAS and to assist the movements of the wearer.

We registered the grasping and reaching motions in the BOTAS system before participants performed any task. To generate time-series data of the BOTAS reaching motion, we selected appropriate movement duration and start and end positions of the BOTAS hand in a plane, including the BOTAS upper arm and forearm. On the basis of these motion parameters, profiles of the end-effector (BOTAS hand) position were calculated based on the minimum jerk model (Flash and Hogan, 1985) or the minimum torque-change model (Uno et al., 1989). Because the latter model requires individual parameters, such as mass, inertia moment, length, and center of gravity of the arm, we used the minimum jerk model in this study.

BOTAS hand positions (*x*(*t*), *y*(*t*)) were calculated by minimizing the following criterion function:

(1) $$J=\int_{0}^{T}\left[{\left(\frac{d^{3}x}{dt^{3}}\right)}^{2}+{\left(\frac{d^{3}y}{dt^{3}}\right)}^{2}\right]\text{​}dt$$

where *T* denotes the movement duration. In the calculation, the positions were constrained so that the velocities and accelerations at the start and end positions would be zero and the positions would not exceed the ROM of each wearer. In cases where the wearer had sufficiently wide ROM, the calculated hand-positions were along the straight line connecting the initial and end positions, as has been shown before for some rehabilitation robots using the minimum-jerk model (Krebs et al., 2003; Amirabdollahian et al., 2007; Wolbrecht et al., 2008). On the other hand, in a case where the user has a narrow ROM, because, for example, of paralysis, and the positions along the straight line violated the ROM constraints, the calculation instead automatically generated positions along a curved line that did not exceed the ROM.

Time-series data for each BOTAS joint were calculated by solving the inverse kinematics of the BOTAS arm. The control signals to the BOTAS joints (*U*_*n*_) were calculated continuously using these time-series data and BOTAS joints were controlled based on the PID algorithm, referring to the error (*e*_*n*_) between the current angle (θ_*n*_) and the desired angle (θ_*dn*_):

(2) $$\begin{array}{c} U_{n}=K_{P}e_{n}+K_{I}\int_{0}^{t_{c}}e_{n}dt+K_{D}\frac{de_{n}}{dt}-R_{n},\\ e_{n}=\theta_{n}-\theta_{dn},\\ R_{n}=\{\begin{array}{c} \lambda_{n}+\tau_{n}(n=1,4)\\ \tau_{n}(n=5,7,8,9) \end{array} \end{array}$$

Here, *n* denotes the joint number (see Figure 2A) and *K*_*P*_, *K*_*I*_, and *K*_*D*_ denote the proportional, integral, and differential gains, respectively, and *t*_*c*_ is the current time. Additionally, *R*_*n*_ represents a correction term that can refer to the cable interference (λ_*n*_) and torque interference (τ_*n*_) when the control signals are calculated.

#### Preparation for data acquisition

To detect SSVEP signals, three electrodes were located at Oz, PO7, and PO8. These electrodes were referenced to C4 and grounded to AFz. Each electrode position was defined based on the 10–10 EEG coordinate system. EEG signals were recorded with an EEG amplifier (g.USBamp, g.tec, Guger Technologies OG, Austria) at 256 Hz. The EEG signals recorded were filtered with an eight-order 5–30 Hz bandpass filter.

#### Visual stimulus devices

To elicit SSVEP, we prepared three LED flickers. The visual stimulus devices have green and blue LEDs, placed in a checkerboard pattern (eight green LEDs and eight blue LEDs in each device) and LEDs of each color flicker alternately. Additionally, an acrylic board was placed above the LEDs. The size of the device was 3 (W) × 3 × (D) 2.5 (H) cm.

### Participants

Twelve able-bodied participants [age: mean (*SD*) = 29.2 (6.2); four females] and three patients with upper cervical SCIs (P1–P3; see Table 3) who had not previously participated in this study were recruited. All able-bodied participants were right-handed. Our study was approved by the Institutional Review Board at the National Rehabilitation Center for Persons with Disabilities. All participants provided written informed consent in accordance with institutional guidelines.

### Experimental paradigm

#### Calibration to permit SVM classification

To classify EEG signals online in BOTAS-assisted trials, we used SVM featuring a radial basis function kernel. SVM is a classification technique based on statistical learning theory (Vapnik, 1996).

For calibration, we recorded EEG signals from PO7, Oz, and PO8 when participants were either fixated or not on the LED flickers. The LED flicker was placed in front of the participants at an 80-cm distance. One calibration trial consisted of a fixation phase with the LEDs flickering for 5 s and a non-fixation phase of 5 s. The target frequencies of LED flickering were 6, 7, and 8 Hz, and each participant engaged in 10 trials frequency.

EEG signals were buffered every 0.1 s for 3 s in each frame and FFT and CCA were used to analyze the buffered signals. We used EEG signals from Oz (only) to construct a feature vector (FV) in each frame because this electrode yielded the highest signal-to-noise ratio (Table 1). Each FV was composed using a combination of values calculated by subtractions of FFT and CCA outputs at target and non-target frequencies. In detail, FV was defined as:

(3) $$\text{FV}=\left[F_{6}\;F_{7}\;F_{8}\;F_{12}\;F_{14}\;F_{16}\;C_{6}\;C_{7}\;C_{8}\;C_{12}\;C_{14}\;C_{16}\right]\text{​}.$$

**Table 1:** **The *P*-values obtained upon paired *t*-testing of the significance of differences in peak values obtained in the non-fixation and fixation phases**.

**Electrode**	**6 Hz**	**7 Hz**	**8 Hz**	**12 Hz**	**14 Hz**	**16 Hz**
PO7	0.55	0.74	0.15	0.38	0.13	0.12
Oz	0.07^+^	0.01^*^	0.07^+^	0.09^+^	0.08^+^	0.008^**^
PO8	0.17	0.78	0.14	0.43	0.06^+^	0.12

+ p < 0.1,

* p < 0.05,

** p < 0.01.

Here,

(4) $$\begin{array}{c} \begin{array}{cc} F_{i}=f(i)-\sum_{j\;\neq \;i}f(j), & C_{i}= \end{array}c(i)-\sum_{j\;\neq \;i}c(j),\\ \begin{array}{cc} F_{2i}=f(2i)-\sum_{j\;\neq \;i}f(2j), & C_{2i}= \end{array}c(2i)-\sum_{j\;\neq \;i}c(2j),\\ (i,j=6,7,8). \end{array}$$

Here, *f*(*i*) and *c*(*i*) denote the spectrum powers at *i* Hz (i.e., the frequency of LED flickering), calculated using FFT and CCA, respectively. *f*(2*i*) and *c*(2*i*) are the values of the second harmonics. On the other hand, *f*(*j*), *c*(*j*), *f*(2*j*), and *c*(2*j*) denote those values at non-target frequencies. To calibrate SVM, we prepared 4 classes: fixation at 6, 7, and 8 Hz and non-fixation, and each feature vector was assigned, respectively. We prepared 400 samples for each class.

#### Performance evaluation of the BOTAS system

**Figure 3:**
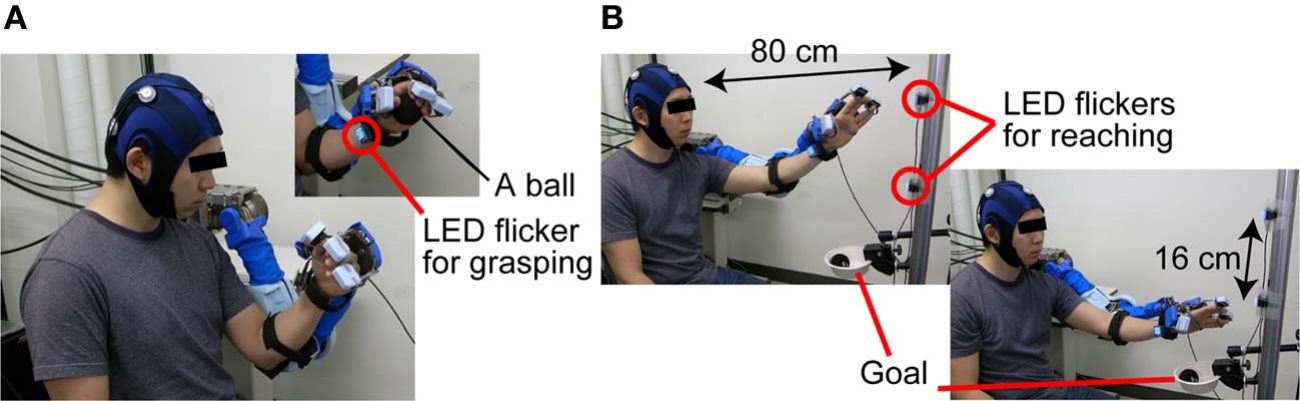
**The sequence used in BOTAS-assisted trials. (A)** To trigger the grasping movement, participants were asked to fixate on the LED flicker on their wrist. **(B)** Then, the participants started to fixate on the LED flicker attached to the poll and started the BOTAS-assisted reaching movement, toward the upper or lower target. The ball was released over the goal position.

For BOTAS-assisted trials, the able-bodied participants sat on an adjustable-height chair and wore the BOTAS on their left arm. P1, P2, and P3 sat in their wheelchairs and the position of the BOTAS arm was adjusted to the patients' left arm. A LED flicker was attached to the BOTAS around the wrist joint (Figure 3A) and two further LED flickers were attached to a pole placed 80 cm from a participant. The distance between these two LED flickers was 16 cm (Figure 3B). We assigned the grasping movement to the LED flicker attached to the BOTAS wrist joint. The LED flickers attached to the pole were assigned reaching movements in the up or down directions. In BOTAS-assisted trials, the left arms of participants were moved passively by BOTAS. One trial consisted of a grasping movement, a reaching movement in the upper or lower direction, and a release movement (hand unclenching). Every trial featured six phases (A–F), as described below.

Phase A: Waiting for the task to start in the initial posture.

Phase B: A participant started to fixate on the LED flicker on the wrist joint when a beep sound was presented as a start signal (Figure 3A).

Phase C: When SVM classified the EEG signal into a specified frequency, a BOTAS-assisted grasping movement was started. The experimenter passed a ball to the participant.

Phase D: After the grasping movement, a beep sound was again presented, and the participant began to fixate on the upper or lower target LED flicker on the pole, to trigger a reaching movement. The reaching target in odd-numbered trials was the upper LED flicker and that in even-numbered trials was the lower LED flicker.

Phase E: When SVM classified the EEG signal into a specified frequency, a BOTAS-assisted reaching movement was started. Then, the ball was released at the goal position by means of BOTAS-assisted finger movements (Figure 3B).

Phase F: Return to the start position.

Each participant was asked to fixate on a LED flicker for 10 s in Phases B and D. All participants repeated the trial (Phases A–F). All participants performed the trial 30 times, except for patient P1 who performed the trial 20 times. The frequencies of LED flickering were randomly changed every 10 trials; for example, 6 Hz (wrist), 7 Hz (upper target), and 8 Hz (lower target) in the first 10 trials; 8 Hz (wrist), 6 Hz (upper target), and 7 Hz (lower target) in the next 10 trials; 7 Hz (wrist), 8 Hz (upper target), and 7 Hz (lower target) in the last 10 trials. Repetitive trials allowed us to explore the robustness of our BOTAS system under asynchronous control. In other words, the dependence of performance on visual stimulus (i.e., location and flickering frequency of a LED flicker) was evaluated.

SVM conducted online classification of the recorded EEG signals (3-s buffered data) every 0.1 s. The SVM classification result was used to determine the LED flicker upon which the participant fixated. After a participant started to fixate on one of the LED flickers during phases B and D, the BOTAS system was sent a control command according to the SVM classification result. In other words, participants could control initiation of a pre-recorded BOTAS motion when gazing at a target LED.

## Results

### Able-bodied participants

#### FFT spectrum power

**Figure 4:**
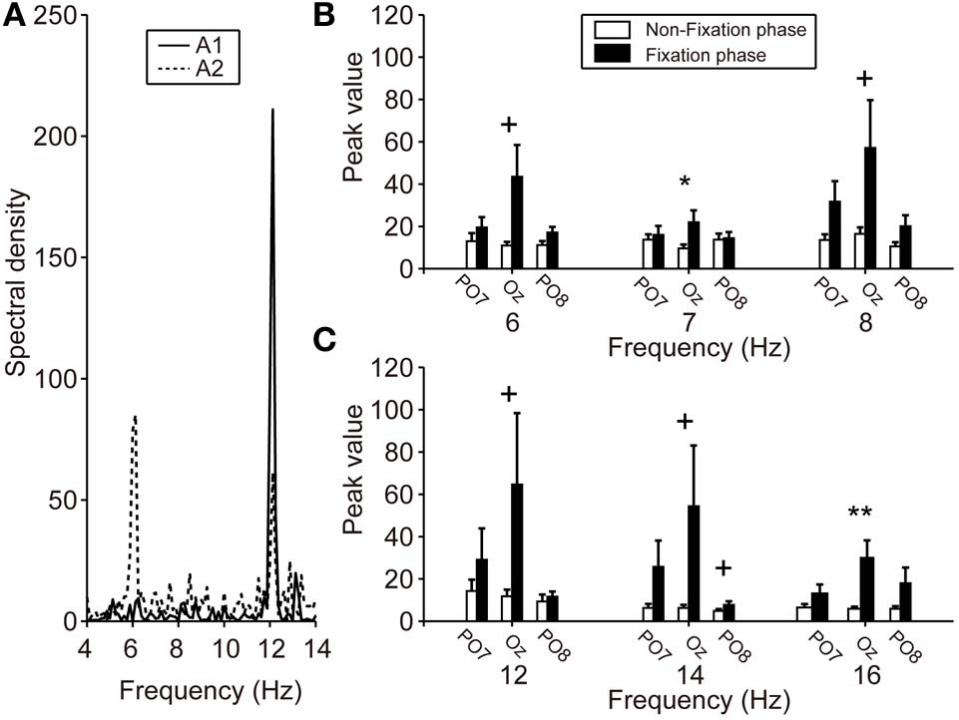
**SSVEP power during calibration. (A)** Particular frequency spectra of EEG signals from Oz while fixating on the LED flicker at 6 Hz. Solid and dotted lines indicate the A1 and A2 results, respectively. The A1 spectrum revealed an increased frequency power at 12 Hz (i.e., a harmonic of 6 Hz), but A2 showed increases at both 6 and 12 Hz. **(B,C)** Mean spectral power of each electrode. Left, center, and right pair bars indicate the peak values of PO7, Oz, and PO8, respectively. Compared with the peak values during the non-fixation phase (white bars), those during the fixation phase (black bars), especially Oz, showed strengthening. Error bars indicate SE across participants. ^+^*p* < 0.1, ^*^p < 0.05, ^**^p < 0.01.

Figure 4A shows typical frequency spectrum results, calculated by averaging EEG signals obtained during calibration (able-bodied participants A1 and A2). In particular, when the participants fixated on the LED flicker at 6 Hz, the EEG power recorded from Oz was increased at 6 and 12 Hz. These changes in the frequency spectrum indicated that the LED flicker could elicit SSVEP. The spectrum of A1 indicates that frequency power became strong at the second harmonic of the LED flicker upon which A1 fixated (i.e., 12 Hz). On the other hand, the frequency power increased not only at the second harmonic, but also at the same frequency as the LED flicker (i.e., 6 Hz) in A2. Because the responses in SSVEP could vary among individuals, the feature vector, shown in (3), included the results of frequency analyses for not only the LED frequencies, but also their second harmonics.

Figures 4B,C show the peak values of FFT powers (means) at the various frequencies of the LED flicker (6, 7, and 8 Hz) and their second harmonics (12, 14, and 16 Hz) during the non-fixation (white bars) and fixation (black bars) phases of calibration. Mean values were calculated from the specific frequency band (target frequency ± 0.125 Hz). Peak values during the non-fixation phase represented the noise level in each channel (white bars in Figures 4B,C). When the participants fixated on the LED flicker, FFT powers increased, compared with those during the non-fixation phase, especially in Oz. Here, we compared the peak values of the non-fixation and fixation phases to select an electrode with the highest signal-noise ratio (SNR). *P*-values from paired *t*-tests comparing peak values in the non-fixation and fixation phases (PO7/Oz/PO8) are listed in Table 1. A small *p*-value is indicative of a high SNR. Statistical testing revealed that Oz was the principal SSVEP signal source (i.e., exhibiting a high SNR), relative to PO7 and PO8. In our efforts to construct a user-friendly BMI system, we sought to use as few electrodes as possible. Therefore, we used the EEG signal from Oz (only) to calibrate SVM and to perform online classification in BOTAS-assisted trials.

#### Classification accuracy

To evaluate the performance of the SSVEP-BMI system, we calculated the classification accuracy of EEG signals in BOTAS-assisted trials. Depending on the first classification into frequency classes (6, 7, or 8 Hz) during the fixation phase (phases B or D), we determined whether the classification in each trial was correct. If SVM first classified the EEG signal into any class other than the target frequency class-for example, despite a participant fixating on the LED flicker at 6 Hz, SVM classified the EEG signals into the 7 or 8 Hz class-the trial was defined as false.

**Figure 5:**
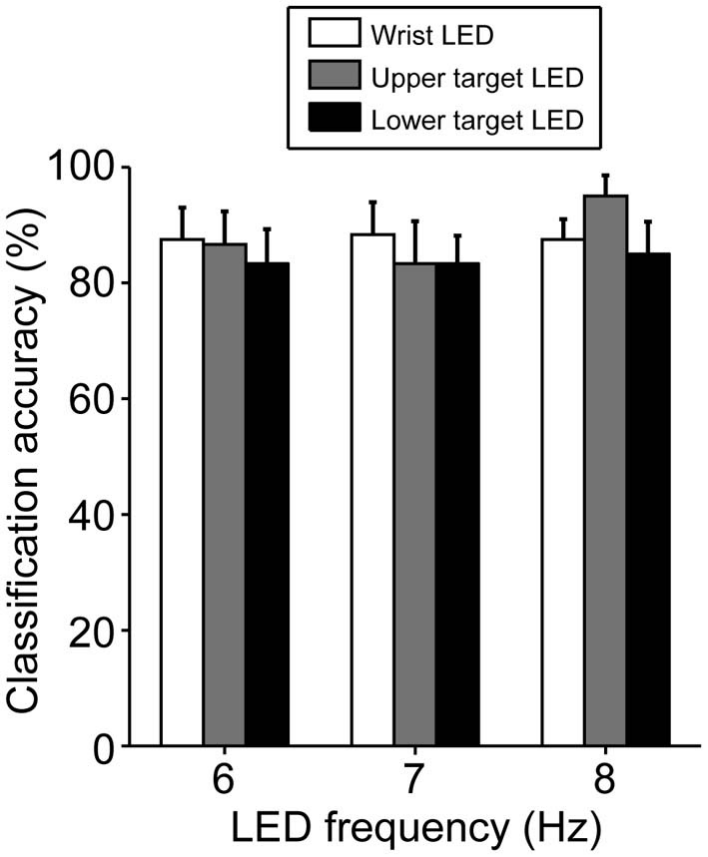
**Classification accuracy in BOTAS-assisted trials based on EEG signals from Oz.** Accuracy was not dependent on the frequency or location of the LED flickers. Error bars indicate SE across participants.

The classification based on SVM was 80–90% accurate, on average, under all LED settings used (Figure 5). Only one participant (A10) yielded a poor classification accuracy (less than 70%, on average, across all LED settings) but most participants (8 of 12) exhibited good performance, with 90–100% classification accuracy (Table 2). To clarify the dependence of LED frequency and location on SVM performance, we performed Two-Way ANOVA (frequency of LED flickering × position of LED flickers). No significant main effect or interaction was apparent [frequency: *F*_(2, 22)_ = 0.49, *p* = 0.62; position: *F*_(2, 22)_ = 0.95, *p* = 0.40; interaction: *F*_(4, 44)_ = 1.36, *p* = 0.26].

**Table 2:** **Individual performances across all LED settings in able-bodied participants**.

	**A1**	**A2**	**A3**	**A4**	**A5**	**A6**	**A7**	**A8**	**A9**	**A10**	**A11**	**A12**
CA (%)	98.3	98.3	80.0	96.7	90.0	91.7	93.3	98.3	75.0	65.0	96.7	78.3
Delay (s)	3.1	2.8	4.3	2.5	2.5	2.3	2.7	2.6	2.9	4.0	2.5	2.8

CA, Classification accuracy.

#### Delay in SSVEP detection

**Figure 6:**
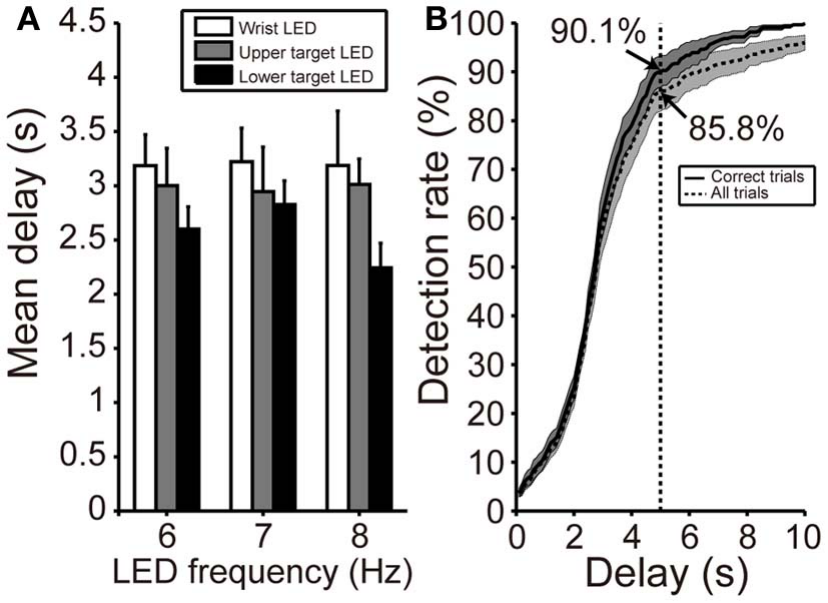
**Delay in initiation of BOTAS movement. (A)** Mean delay from initiation of fixating on the LED flicker to driving BOTAS. These results were calculated based on correct trials. The delay was of slightly longer duration when the LED was attached to the participant's wrist. Error bars indicate SE across participants. **(B)** SSVEP detection rate after fixation on the LED flicker (*i.e*., phases B and D). The results indicated by solid and dotted lines represent data calculated from correct trials and all trials, respectively. The BOTAS system began to detect SSVEP 2 s after initiation of fixating on the LED flicker. Participants successfully initiated grasping or reaching motions within 5 s in more than 85% of all trials.

Figure 6A shows the mean delay in SVM classification after participants fixated on any LED flicker in phases B or D. These delays indicate the time from LED fixation to driving of BOTAS. The results in Figure 6A are evaluation of only correct trials. The proportion of correct trials with respect to all trials was 88.5%. Using the LED setting associated with the shortest delay (frequency of LED flickering: 8 Hz, position of the LED flicker: lower target), SVM required about 2 s to classify the EEG correctly. At other LED settings, SSVEP also functioned correctly in less than 3 s. To clarify the dependence of LED frequency and location on SVM performance, repeated Two-Way ANOVA (frequency of LED flickering × position of the LED flickers) was used to analyze the delays (Figure 6A). ANOVA indicated that only the position of the LED flickers was significant [frequency: *F*_(2, 22)_ = 0.23; *p* = 0.79; position: *F*_(2, 22)_ = 4.35, *p* < 0.05; interaction: *F*_(4, 44)_ = 0.45, *p* = 0.77]. Additional analysis revealed a significant difference between the wrist and lower target LEDs (*p* < 0.05, Bonferroni test).

When participants fixated on any LED flicker during phases B or D, the detection rate increased with time (Figure 6B). The solid line indicates the detection rate in correct trials and the dotted line in all trials, included false trials. At 2 s after fixating, the detection rate increased sharply and the SVM classification for the grasping or reaching movement was success, 90.1% of correct trials and 85.8% of all trials within 5 s. Individual delays are shown in Table 2.

### Patients with upper cervical SCI

Patients in this study did not have spasticity in the left arm; however, their arm joints showed narrower ROMs compared with the able-bodied participants. When patients participated in this task, we defined the task space based on the limited ROM.

Table 3 shows the SVM performances of three patients with upper cervical SCI. Because Oz impedance in P1 did not decrease over time, the calibration and BOTAS-assisted trials featured high impedance. Classification accuracy and mean delay were slightly lower performance vs. those of the able-bodied participants. Accuracies were not less than 80% and delays were shorter than 4 s. We confirmed that the patients with upper cervical SCI operated the BOTAS system successfully. They successfully grasped the ball and transferred it to the goal position in a high proportion of trials (P1: 18/20 trials, P2: 29/30 trials, P3: 28/30 trials). No patient reported discomfort during task performance.

**Table 3:** **Performance of patients in BOTAS-assisted trials**.

**Participant (Age, Gender, Time since injury, Injury level)**	**Classification accuracy (%)**	**Mean delay (s)**	**Detection rate within 5 s (%)**
P1 (42, M, 16y, C6)	80.0	3.8	77.5
P2 (40, M, 19y, C3)	83.3	3.9	73.5
P3 (51, M, 24y, C6)	80.0	3.7	82.3

## Discussion

We prepared life-size robot arms BOTAS that can assist the wearer's goal-directed movements of the upper limb, such as reaching or grasping. To control the motion of the BOTAS, we recorded EEG signals. SSVEP was elicited, especially from Oz, during fixation on a LED flicker. In BOTAS-assisted trials, both able-bodied participants and patients with upper cervical SCIs successfully controlled the grasping-a-ball and carrying-the-ball movements in a high proportion of trials.

### Asynchronous control of goal-directed movements

We developed the SSVEP-based BMI assist suit for the whole arm and fingers to support goal-directed actions involving multiple body parts, so that the devices could be used for movements such as those involved in OT training. Goal-directed activity has greater success in helping patients with paresis organize their movements effectively, compared with an exercise with no goal (Ma and Trombly, 2002; Pillastrini et al., 2008). Previous studies made use of rehabilitation robots with relatively high DOFs for shoulder and elbow motions (Sanchez et al., 2006; Ball et al., 2009; Dolce et al., 2009; Staubli et al., 2009) or finger motions (Schabowsky et al., 2010). However, providing a useful series of actions, such as reaching and grasping, was not easy using these robots. In this study, both able-bodied participants and patients with upper cervical SCIs successfully performed the grasping-a-ball and carrying-the-ball movements, which require not only shoulder and elbow motions but also wrist and finger motions, thus representing a purposeful and goal-directed movement. The effectiveness of movements used in rehabilitation training must be studied further, but our BOTAS system is suggested to be potentially useful for rehabilitation of patients with upper limb disabilities. In terms of clinical evaluation, it would be wise to evaluate user satisfaction (e.g., by applying the Quebec instrument evaluating satisfaction with assistive technology; QUEST 2.0) (Zickler et al., 2011).

In rehabilitation training using BMI technologies, an artificial closed-loop between the brain and the impaired body part(s) facilitates brain plasticity (Lebedev and Nicolelis, 2006; Gomez-Rodriguez et al., 2011). Additionally, synchronization between user intent and the action of the external device is important in BMI-based rehabilitation training. Recent invasive BMI technologies have succeeded in the asynchronous control of robot arms for useful series of actions, such as reaching and grasping (Hochberg et al., 2012). Several studies have used non-invasive BMI technologies to control assistive robots according to user intent (Muller-Putz and Pfurtscheller, 2008; Horki et al., 2010, 2011; Pfurtscheller et al., 2010b; Ortner et al., 2011). In this study, we prepared a pre-recorded series of useful actions-a grasping-a-ball movement and a carrying-the-ball movement—and provided asynchronous control using SSVEP signals. A SSVEP signal was used to trigger the grasping-a-ball movement and another SSVEP signal was used to trigger the carrying-the-ball movement. Although we did not attempt to directly decode user intention, participants fixated on LED flickers when they wished to start movement. Also, the hand and arm were visible when movements were made; this may have contributed to closed-loop sensory feedback. Asynchronous BMI systems using SSVEP may be useful for closed-loop rehabilitation approaches that make use of repetitive movement tasks (Horki et al., 2010; Diez et al., 2011; Ortner et al., 2011). Recent studies have further suggested that synchronization enabled by BMI between “motor intention of a wearer” and “motion of external device” render rehabilitation training effective (Ramos-Murguialday et al., 2013). In the BOTAS system, a wearer fixates on a LED flicker when she/he wants to drive motion, and BOTAS then comes into play. Thus, motor intention and BOTAS motion are synchronized. Previous studies suggest that our system will be effective in rehabilitation training, although further work is needed.

### SSVEP features in the BOTAS system

To construct a user-friendly BMI system, it is important that EEG signals are recorded using only a few electrodes (Luo and Sullivan, 2010). Many BMI systems in previous studies used multiple electrodes to detect SSVEP (Muller-Putz and Pfurtscheller, 2008; Horki et al., 2011; Ortner et al., 2011). Although use of multiple electrodes may facilitate detection of EEG signals and increase classification accuracy (Bin et al., 2009; Grave De Peralta Menendez et al., 2009; Bakardjian et al., 2010), multiple electrode placement requires considerable time, may burden users, and may be difficult to apply in rehabilitation training. Thus, practical BMI systems using small numbers of electrodes are potentially useful and may reduce user discomfort (Zickler et al., 2011). When recording EEG signals with a few electrodes, brain areas in which SSVEP is strongly induced should be focused on exclusively. SSVEP was not strong in lateral areas (for example, PO7 and PO8). Placement of electrodes in the central area, such as Oz, ought to be effective for SSVEP-BMIs (Pastor et al., 2003; Bin et al., 2008, 2009). Indeed, we found that the classification accuracy was over 80% using the EEG signal from Oz alone, but it would be valuable to further improve classification accuracy and decrease delay by optimizing the signal processing software and visual stimuli (i.e., hardware).

The colors and frequencies of visual stimuli are also important parameters for effective elicitation of SSVEP. Takano et al. (2009) reported that green/blue flicker stimuli improved EEG signal classification accuracy and the usability of the P300-based BMI system, compared with white/gray stimuli. This color tuning should also be effective in SSVEP-based BMIs. Further, SSVEP is strongly elicited at frequencies below ~20 Hz (Pastor et al., 2003; Bakardjian et al., 2010), and low-frequency LED flickers worked well in this study. Further work on optimization of visual stimuli is required.

The delay in SSVEP detection was affected by the LED location (wrist vs. target position). The delay was longer when the participants fixated on the LED flicker attached to their wrist, than in the other locations. Participants were asked to fixate on a LED flicker placed 80 cm away to yield EEG signal data permitting SVM calibration. Because the distance from the eyes of participants to the wrist-attached LED was ~40 cm, variation in the experimental setting (i.e., the location of LED flickers) will likely change perceived stimulus intensity or viewing angle, thus affecting the SSVEP response. On the other hand, the classification accuracies of the EEG signals from Oz did not depend on LED frequency or position. Thus, our BOTAS system exhibited robustness in terms of EEG classification and allowed the LED parameters (frequency, location) to be set according to the task or environment.

In this study, the participants were able to control BOTAS successfully using SSVEP. The system could be operated with little training and BOTAS could be driven asynchronously whenever the wearer wished to. EEG signals recorded from the visual cortex (Oz) were used in classification. The data indicate that our BOTAS system is potentially useful in rehabilitation of patients with upper limb disabilities. Future work, including unit downsizing, will allow us to develop an intelligent orthosis useful in terms of daily life support.

### Conflict of interest statement

The authors declare that the research was conducted in the absence of any commercial or financial relationships that could be construed as a potential conflict of interest.
